# Molecular Epidemiological Study of Pyrazinamide-Resistance in Clinical Isolates of *Mycobacterium tuberculosis* from South India

**DOI:** 10.3390/ijms11072670

**Published:** 2010-07-07

**Authors:** Muthuraj Muthaiah, Sridharan Jagadeesan, Nisha Ayalusamy, Manupriya Sreenivasan, Sambamurthy Sangamesvara Prabhu, Usharani Muthuraj, Kamatchiyammal Senthilkumar, Saroja Veerappan

**Affiliations:** 1 State TB Training and Demonstration Centre, Govt. Hospital for Chest Diseases, Puducherry, India; 2 Departments of Microbiology, Vector Control Research Centre, Puducherry, India; 3 Virology Cell, NEERI, CSIR Complex, Tharamani, Chennai, Tamil Nadu, India

**Keywords:** PZA resistance, mycobacterium tuberculosis, pulmonary tuberculosis, South-India, pncA mutation

## Abstract

Pyrazinamide (PZA) has been in use for almost 50 years as a first-line drug for short-course chemotherapy against *Mycobacterium tuberculosis.* In this study, PCR mediated automated DNA sequencing is used to check the prevalence of PZA resistance among treatment failure cases of pulmonary tuberculosis. Out of 50 clinical isolates examined, 39 had mutations in the pncA gene that encodes Pyrazinamidase, an enzyme required to activate PZA. Of these, 31 (79.5%) were localized to three regions of pncA. We found two isolates with hitherto unreported mutation at amino acid 26 (Ala→Gly) of pncA.

## 1. Introduction

Pyrazinamide (PZA); a nicotinamide analog, has been in use for almost 50 years as a first-line drug for short-course chemotherapy against *Mycobacterium tuberculosis*. PZA is bactericidal to semidormant mycobacteria and reduces the total tuberculosis treatment time when used in combination with isoniazid and rifampin. This has made PZA the third most important drug in the list of modern therapy for tuberculosis [1]. PZA plays a unique role in achieving this shortened therapy, because PZA appears to kill at least 95% of the semi-dormant bacterial population persisting in a low-pH environment, since its activity is present only in the acidic environment found in active inflammation [2].

Pyrazinamide is a prodrug that must be activated by a bacterial pyrazinamidase (PZase) enzyme to the active form pyrazinoic acid (POA), which is toxic to *M. tuberculosis*. Although a specific target for POA remains unknown, it has been suggested that POA accumulation results in a pH reduction which, in turn, leads to a non-specific inhibitory effect on cellular metabolism [3,4]. Recently, it has been shown that POA can disrupt *M. tuberculosis* membrane potential, affecting the transport function at an acidic pH. Pyrazinamidase (PZase) is a non-essential enzyme encoded by the pncA gene. Mutations in the pncA gene causing pyrazinamide resistance have been well characterized [5] and are located along the entire pncA gene open reading frame as well as in its putative regulatory region [6]. These mutations lead to loss of PZase activity, and hence the mutant will not be able to activate pyrazinamide to form POA.

Conventional pyrazinamide susceptibility testing by agar proportion or by Lowenstein–Jensen proportion method is labor intensive and may exhibit high discordance rates among different laboratories as the media pH and other parameters are known to influence the outcome of the susceptibility testing [7]. Automated systems, such as the BACTEC 460 TB system, BACTEC MGIT 960 are commercially available, but are expensive and impractical to use in developing countries where the prevalence of tuberculosis is high. Many earlier studies have shown that inactivation of the pncA gene by mutations is the major mechanism for resistance to PZA [8]. This study was designed to determine the frequency and the distribution of the pncA mutations among failed treatment cases of pulmonary tuberculosis in South India. *In vitro* susceptibility to PZA was correlated with PZase activity and the pncA nucleotide sequence.

## 2. Results and Discussion

**Figure 1.**
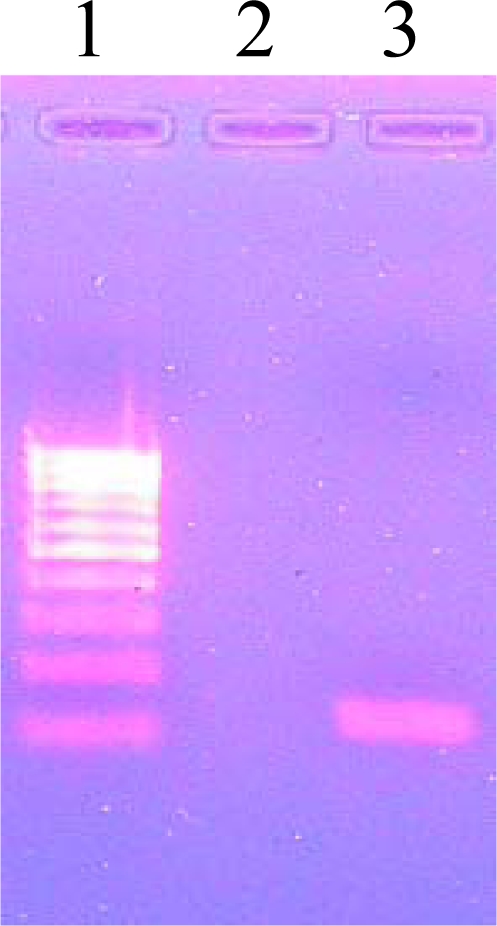
PCR Amplification for Species Identification. Lane 1: 100 bp DNA ladder, Lane 3:123 bp PCR amplified product.

**Figure 2.**
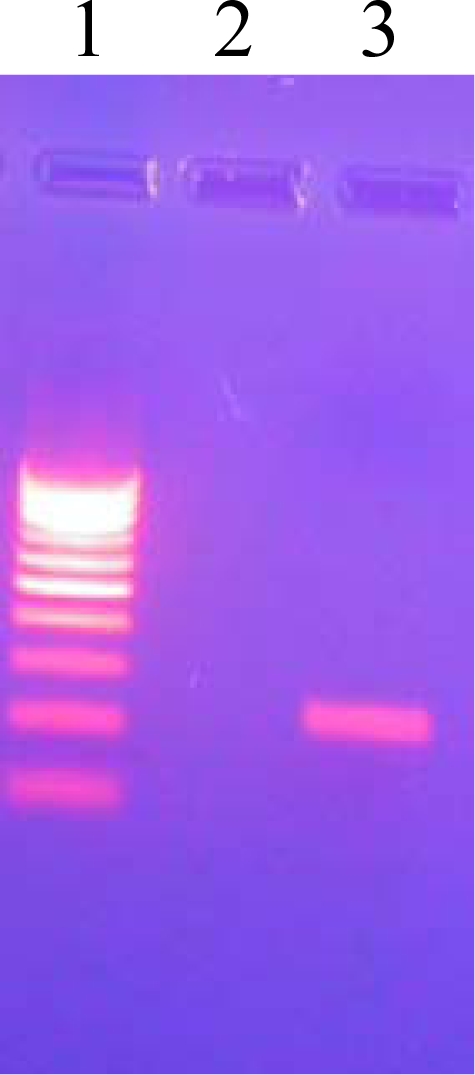
PCR Amplification of pncA. Lane 1: 100 bp DNA ladder, Lane 3: 222 bp PCR amplified pncA gene product.

**Figure 3.**
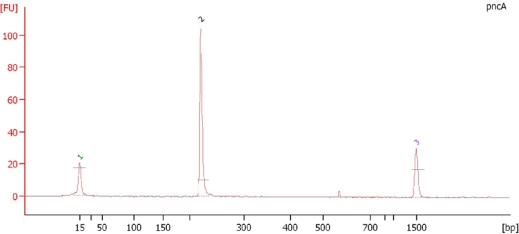
Electropherogram Analysis of PCR Amplified Products. Peak 1: Lower Marker (15 bp), Peak 2: PCR amplified product of pncA gene (222 bp) and Peak 3: Upper marker (1500 bp).

A clear PCR product band of 123 base pairs (bp) was observed on a 2% agarose gel confirming the *M. tuberculosis* (Figure 1).The observation of a clear band at the 222 bp region on 2% agarose gel confirmed the amplification of the pncA gene of *M. tuberculosis* (Figure 2).The PCR products were analyzed on a Bioanalyzer (Agilent 2100) to check the purity and specificity of the products. Electropherogram analysis of the PCR-amplified pncA gene confirmed the molecular size (222 bp) of the products (Figure 3).

We analyzed 50 clinical isolates of *M. tuberculosis* from treatment failure cases to find mutations in the pncA gene. *In vitro* susceptibility testing showed that 39 of these isolates were resistant to PZA with minimal inhibitory concentrations (MIC) of 300 μg/mL, and 11 out of 50 were PZase positive and susceptible to PZA with a minimal concentration of 100 μg/mL (Table 1). The results of the sequence analysis of pncA from 39 PZA-resistant isolates are presented in Table 2. We found nucleotide substitutions and insertions leading to change in the amino acids. The nature of the pncA mutations includes substitutions of amino acids (33 of 39 totals PZA-resistant isolates with pncA *mutations*), insertions causing nonsense peptides (6 of 39 isolates). The distribution of pncA mutations is dispersed along the gene. The reason for treatment failure in the 11 PZA sensitive cases could be due to some other mechanisms. The susceptible clinical *M. tuberculosis* isolates were found to have identical wild-type sequence, while 75–95% of PZA resistant isolates had a point mutation (mis-sense/nonsense or insertion/deletion) and these were spread over the entire length of the pncA gene [9–11] It is generally considered that mutations leading to PZA resistance are scattered along the pncA gene [12,13]. However, some authors have mentioned a certain degree of conservation of pncA mutations at amino acid residues 3 17, 61–76 and 132–142 in the PZase protein [14–16]. We found that 35.9% of the mutations are located in regions that are different from those reported previously [17]. Mutations were observed in the regions between residues 132 and 142, and in 33.3% between residues 69 and 72, in 15.4% and 15.4% of the mutations were found between residues 5 and 12. Among the PZA resistant isolates, 39 (41%) exhibited 16 different changes in pncA nucleotide sequence (Table 1). The mutations in pncA included thirteen nucleotide substitutions causing amino acid change in 33 isolates, mutations caused by three insertions in six isolates. In this study we have not observed the relationship between pyrazinamide MIC values and mutation frequency or position. However, it was observed that every isolate that presented a pncA alteration lacked PZase activity (Table 2). Prior studies have shown that common mutations in the pncA are located in three regions, 3–17, 61–85 and 132–142 [2]. In this study we find that one third of the mutations are distributed in the 132–142 region and 30.7% of mutations from 12 isolates are located in the 61–85 region. The region 3–17 accounts for mutations in 6 (15%) of the resistant isolates. Overall, these three regions alone contribute to drug resistant mutations in 79.5% of the isolates. These three regions are important in the formation of the active site of the enzyme [2]. Three isolates showed mutations at K96 and two isolates were mutated at H51. K96 and H51 are also important at the active site and earlier studies have reported mutations at these two sites [2]. We found one isolate mutated at A171. This mutation has been reported in a prior study from Portugal [6]. In our study, we found two isolates (5.1%) showing a unique mutation at A26. The G→C nucleotide changes of both strains led to substitution of a nonpolar amino acid Alanine to another nonpolar amino acid Glycine (Ala→Gly). This mutation has not been reported earlier and the significance of this mutation on PZase activity is not clear. Eleven clinical isolates carried the wild-type pncA sequence and retained PZase activity. This finding supports the hypothesis that other mechanisms may be involved in PZA resistance—possibly alteration in Pyrazinamide uptake, increased POA active efflux [18] or mutations leading to the modification or amplification of an unknown POA target [19].The resistant pattern of clinical isolates due to mutations detected by PCR mediated DNA sequencing were matched with that of *in vitro* sensitivity tests done on L.J. slants (Table. 1). This suggests that mutations in the pncA gene are indicative of PZA resistance, and the importance of PCR mediated pncA gene sequencing in detecting PZA resistance is established.

The present study is one of the first studies on pncA gene mutations in clinical isolates of *M. tuberculosis* in South India. We found that PCR mediated gene sequencing is an effective method for reliable identification of PZA resistance. This study suggests that the enzymatic activity is very sensitive to sequence alterations in any protein region of the pncA gene and most of the PZA-resistant *M. tuberculosis* strains have mutations in the pncA gene, which has implications for developing a rapid test for detecting PZA-resistant *M. tuberculosis* strains. The diversity of methods currently used in clinical laboratories for the detection of PZA resistance in *M. tuberculosis* isolates causes inconsistent results of PZA susceptibility testing [20]. Inconsistent results of PZA susceptibility testing have been reported by a number of laboratories by various methods, including the qualitative BACTEC test [21]. On the basis of our analyses of the PZA-resistant clinical isolates, there is a very good correlation between the loss of PZase activity and pncA mutations and PZA resistance. These findings are the basis for designing PCR mediated DNA sequencing tests to rapidly detect pncA mutations as a correlation of PZA resistance. Analysis of the pncA sequence has found that five PZA-resistant strains determined by the conventional method are in fact susceptible, indicating that the sequence-based test, e.g., direct sequencing by PCR, may be more accurate or reliable. We have demonstrated in this study that pncA mutations in PZA-resistant strains can be readily detected by the PCR mediated DNA sequencing technique. Thus, detection of pncA mutations by direct sequencing is not only fast, but also will avoid the problems of current PZA susceptibility testing. The diversity of mutations makes it unlikely that a suitable molecular method could be devised for rapidly determining the resistance to pyrazinamide in clinical *M. tuberculosis* isolates. This could be useful for directing the treatment of tuberculosis, reducing treatment costs, and potentially limiting the spread of drug-resistant *M. tuberculosis* isolates. The results presented in this study contribute to the knowledge of the molecular mechanisms and epidemiology of pyrazinamide resistance in South India as well as to expanding the known profiles of pncA mutations worldwide. The PCR mediated direct DNA sequencing method seems to have great potential and needs further evaluation for rapid determination of *M. tuberculosis* susceptibility to pyrazinamide.

## 3. Experimental Section

### 3.1. Bacterial Samples

A total of 50 clinical strains of *Mycobacterium tuberculosis* were isolated from sputum samples of treatment failure cases hospitalized at State TB Training and Demonstration Centre (Intermediate Reference Laboratory) Government Hospital for Chest Diseases, Puducherry, South India, during the period from January 2006 to November 2008. Among the 50 sputum samples collected, 32 samples were from patients of Puducherry state and 18 samples were from the other neighboring border states of Puducherry in India. All specimens were processed immediately and aliquots of the decontaminated specimens were kept at −20 °C

### 3.2. Sputum Processing for AFB Culture

To each volume of sputum, 2 volumes of 4% NaOH were added. The bottles were shaken by hand for 1 minute. Then the bottles were placed in a rack on the shaking machine and were left to shake gently for 20 minutes. The specimens were removed from the shaker. The sputum bottles were centrifuged for 15 minutes at 4000 rpm. After the bottles were removed from the centrifuge, the supernatant was carefully poured off into the disinfectant bath. The bottles were filled with 20 mL of sterile distilled water, shaken by hand to mix the deposit and were centrifuged for 15 minutes at 4000 rpm. The supernatant was poured off and finally the sediment was inoculated with a 5 mm diameter loop onto the pre-sterilized and numbered Lowenstein-Jensen’s slopes. The inoculated media was placed in the 37 °C incubator [22,23].

### 3.3. Drug Susceptibility Test-Proportion Method (Stand and Economic Variant)

With a loop, a representative sample of approximately 4–5 mg is taken from the primary culture and placed in a McCartney bottle containing 1 mL sterile distilled water and 3 mm diameter of 6 glass beads. The bottle was vortexed for 20–30 seconds and the opacity of the bacterial suspension was then adjusted by the addition of distilled water to obtain a concentration of 1mg/mL of tubercle bacilli by matching with McFarland standard No.1. After preparing the standard neat suspension, the dilution 10^−2^ dilution 10^−4^ were produced by discharging two loopfuls (24 SWG-3 mm Nichrome wire) of the bacterial suspension. The contents were mixed by shaking. Two slopes of medium without drug, and two slopes of acidified L.J media are used as controls for the test, and one slope of medium with Pyrazinamide drug (100 μg/mL) are inoculated with a loopful of each dilution. The slopes were incubated at 37 °C and the proportion tests were read at 28 days and again at 42 days [24].

### 3.4. PZase Assay

The PZase assay was performed by the method described in the Clinical Microbiology Procedure Handbook [25]. Briefly, 6.5 g of Dubos broth base, 0.1 g of PZA, 2.0 g of sodium pyruvate and 15.0 g of agar were dissolved in 1 L of distilled water and heated to dissolve the components. The solution was dispensed in 5-mL amounts into screw-cap tubes and stored at 2 to 8 °C until use after solidification of the agar with the tubes in an upright position. A heavy loopful of growth from an actively growing subculture was inoculated. After incubation at 37 °C for 4 or 7 days, 1 mL of freshly prepared 1% ferrous ammonium sulfate was added to each tube. A pink band in the agar indicated a positive test.

### 3.5. Mycobacterium DNA Extraction

One loopful of culture was homogenized in 100 μL of sterile distilled water. The entire homogenized samples were treated with 50 μL of lysozyme (10 mg/mL) at 37 °C for overnight incubation. 70 μL of 14% SDS and 6 μL of Proteinase K (10 mg/mL) was added and incubated at 65 °C for 15 minutes. 10 μL of 5 M NaCl and 80 μL of CTAB/NaCl were added and was incubated at 65 °C for 10 minutes. 800 μL of Phenol: Chloroform: Isoamylalcohol (25:24:1) mixture was added and centrifuged at 10,000 rpm for 10 minutes. The supernatant was transferred to a fresh tube and 600 μL of Isopropanol was added to precipitate the DNA and incubated overnight at −20 °C.Centrifuged at 12,000 rpm in 4 °C for 10 minutes. The pellet was washed with 70% ethanol to remove any remaining solutes. The pellet was air-dried and was dissolved in 20 μL of 1× TE buffer [26].

### 3.6. PCR Amplification for Species Identification

The isolated template DNA was amplified using IS6110 primer in an authorized thermal cycler (Eppendorf Gradient Cycler). This confirmed that the template DNA of the clinical isolates was *Mycobacterium tuberculosis*. The PCR reaction was set up as follows using the primer for *Mycobacterium IS6110* amplification F 5′GTGAGGGCATCGAGGTGG 3′ (10 pmol/μL) R 5′CGTAGGCGTCG GTCACAAA 3′ (10 pmol/μL) [27]. The PCR cycling parameters were 94 °C for 5 minutes; followed by 40 cycles of 94 °C for 1 minute, 57 °C for 1 minute and 72 °C for 1 minute; and a final extension of 72 °C for 10 minutes. The PCR was then kept at hold at 4 °C for 15 minutes. The amplified PCR product was withdrawn from thermal cycler and run on a 2% Agarose gel in TAE buffer. The Ethidium bromide stained gels were observed in a UV Transilluminator and photographed using a Geldoc.

### 3.7. PCR Amplification of *pncA*

The isolated template DNA was amplified using *pncA* primers (P_1_ 5′GTCGGTCATGTTCGCGATCG, and P_2_ 5′TCGGCCAGGTAGTCGCTGAT) [28] in an authorized thermal cycler (Eppendorf Gradient Cycler). The PCR cycling parameters were 94 °C for 5 minutes; followed by 40 cycles of 94 °C for 1 minute, 57 °C for 1 minute and 72 °C for 1 minute; and a final extension of 74 °C for 10 minutes. The PCR was then kept at hold at 4 °C for15 minutes. The amplified PCR product was withdrawn from thermal cycler and run on a 2% Agarose gel in TAE buffer. The Ethidium bromide stained gels were observed in a UV Trans illuminator and photographed using a Geldoc.

### 3.8. Agarose Gel Electrophoresis

The gel running tray was placed in a clean gel casting tray to form the gel uniformly and the comb was fixed at one end. 400 mg of agarose (2%) powder was added to 20 mL of 0.75 × TAE and was boiled to dissolve the agarose completely. Less than 1 μL of Ethidium bromide (0.5 mg/mL) was added into the hand bearable heat 250 mL conical flask containing melted agarose gel and was poured into the gel running tray. 1 μL of gel loading dye was transferred into a 5 × 5 cm size Para film. To it, 5 μL of polymerized DNA was added and mixed thoroughly. The whole volume aliquot of amplified sample with gel loading dye was loaded into a well of 2% agarose gel in 0.75 × TAE buffer and was subjected to electrophoresis for 30 minutes at 100 volts. The gel was observed under UV Transilluminator for specific DNA bands and was photographed. The DNA bands were identified according to size by comparing with the molecular weight marker (100 bp DNA ladder) loaded in a separate lane.

### 3.9. Electropherogram Analysis of PCR Amplified Products

DNA dye concentration and DNA gel matrix were allowed to equilibrate at room temperature. 25 μL of dye concentration was added to DNA gel matrix, vortexed and transferred to spin filter and centrifuged at 2240 g for 15 minutes. The gel dye was allowed to settle at room temperature for 30 minutes. A new DNA chip was placed on the chip priming station. 9 μL of gel dye mix was pipetted into the well marked as G and the chip priming station was closed. The plunger was pressed down until it is held by the chip for 60 seconds. After 5 seconds the plunger was pulled back slowly to 1 mL position. The chip priming station was opened and 9 μL of gel dye was pipetted into the well marked G and 1 μL of ladder was added to the well labeled ladder. 5 μL of marker was pipetted into all 12 sample wells and in ladder well. 1 μL of sample was added into the well. The chip was placed in the Laser Induced Fluorescent instrument (Bioanalyzer-Agilent 2100) and the results were interpreted [29].

### 3.10. DNA Sequencing

The amplified PCR product pncA gene from clinical isolate strains were run on 2% Agarose gel and the PCR product purified using PCR purification kit (Invitrogen). The purified PCR product was directly sequenced in an automated DNA Sequencer at Bioserve in Bangalore. The nucleotide sequence obtained was analyzed using BLASTn Bioinformatics tool available at National Center for Biotechnology Information [30] to know the specificity of PCR amplification and to identify the nucleotide variation. The sequence was further subjected for BLASTx to identify the amino acid changes in comparison with the wild type *Mycobacteruim tuberculosis* (H_37_Rv).

## 4. Conclusions

The worldwide emergence and spread of multi drug-resistant (MDR) strains of *Mycobacterium tuberculosis* has adverse effects on tuberculosis (TB) control programs. The goal of this paper is to describe the advances made in the understanding of the molecular basis of *M. tuberculosis* resistance to PZA, and to discuss the potential of molecular methods in early diagnosis of PZA drug-resistant TB. In the present study, out of 50 clinical isolates examined, 39 had mutations in the pncA gene. Of these, 31 (79.5%) were localized to three regions of pncA. We found two isolates with hitherto unreported mutation at position 26 (Ala→Gly) of pncA. We observed that the 39 clinical isolates had a pncA gene alteration and lacked PZase activity. The reason for treatment failure in the 11 PZA sensitive cases could be due to some other mechanisms. This suggests that the enzymatic activity is very sensitive to sequence alterations in any protein region. Our finding that most PZA-resistant *M. tuberculosis* strains have mutations in the pncA gene has implications for developing a rapid test for detecting PZA-resistant *M. tuberculosis* strains.

## Figures and Tables

**Table 1.** Comparison of *in vitro* testing (on isolates) with PCR mediated direct sequencing.

**PCR Mediated Direct sequencing**	***In vitro* sensitivity tests on L.J. slants (μg/mL)**			
	**Susceptibility (MIC = 100)**	**Resistance**		**PZase activity**
		**MIC = 300**	**MIC > 300**	
Resistant 39 (78%)	00	26(52%)	13(26%)	Negative 39 (78%)
Sensitive 11 (22%)	11(22%)	--	--	Positive 11 (22%)
Total 50 (100%)	11(22%)	39(78%)		Total 50 (100%)

**Table 2.** pncA nucleotide and amino acid changes in PZase–negative *M. tuberculosis* clinical isolates.

**Mutation Site**	**Nucleotide Changes**	**Amino Acid Changes**	**No. of Isolates**	**Percentage (%)**
137	A→C	His→Pro	2	5.1
138	G→A	Cys→Tyr	2	5.1
139	G→C	Val→Leu	2	5.1
142	C→T	Thr→Met	2	5.1
5	AT insertion	Ile→Ser	2	5.1
12	A→C	Asp→Ala	4	10.3
26	G→C	Ala→Gly	2	5.1
51	C→G	His→Gln	2	5.1
69	C→G	Pro→Arg	3	7.7
72	T→C	Cys→Arg	3	7.7
85	T→C	Leu→Pro	6	15.4
96	A→C	Lys →Asn	37.7	
132	TC insertion	Gly→Ser	3	7.7
141	C→A	Gln→Pro	1	2.6
142	AG insertion	Thr→Lys	1	2.6
171	G→C	Ala→Pro	1	2.6
Total			39	100
